# Phylogenetics and biogeography of the two‐wing flyingfish (Exocoetidae: *Exocoetus*)

**DOI:** 10.1002/ece3.2786

**Published:** 2017-02-12

**Authors:** Eric A. Lewallen, Andrew J. Bohonak, Carolina A. Bonin, Andre J. van Wijnen, Robert L. Pitman, Nathan R. Lovejoy

**Affiliations:** ^1^ Department of Biological SciencesUniversity of Toronto ScarboroughTorontoONCanada; ^2^ Departments of Biochemistry & Molecular Biology and Orthopedic SurgeryMayo ClinicRochesterMNUSA; ^3^ Department of BiologySan Diego State UniversitySan DiegoCAUSA; ^4^ University of St. ThomasSt. PaulMNUSA; ^5^ Southwest Fisheries Science CenterNational Marine Fisheries ServiceNational Oceanic and Atmospheric AdministrationLa JollaCAUSA

**Keywords:** cryptic speciation, epipelagic, species delimitation

## Abstract

Two‐wing flyingfish (*Exocoetus spp*.) are widely distributed, epipelagic, mid‐trophic organisms that feed on zooplankton and are preyed upon by numerous predators (e.g., tunas, dolphinfish, tropical seabirds), yet an understanding of their speciation and systematics is lacking. As a model of epipelagic fish speciation and to investigate mechanisms that increase biodiversity, we studied the phylogeny and biogeography of *Exocoetus*, a highly abundant holoepipelagic fish taxon of the tropical open ocean. Morphological and molecular data were used to evaluate the phylogenetic relationships, species boundaries, and biogeographic patterns of the five putative *Exocoetus* species. We show that the most widespread species (*E. volitans*) is sister to all other species, and we find no evidence for cryptic species in this taxon. Sister relationship between *E. monocirrhus* (Indo‐Pacific) and *E. obtusirostris* (Atlantic) indicates the Isthmus of Panama and/or Benguela Barrier may have played a role in their divergence via allopatric speciation. The sister species *E. peruvianus* and *E. gibbosus* are found in different regions of the Pacific Ocean; however, our molecular results do not show a clear distinction between these species, indicating recent divergence or ongoing gene flow. Overall, our phylogeny reveals that the most spatially restricted species are more recently derived, suggesting that allopatric barriers may drive speciation, but subsequent dispersal and range expansion may affect the distributions of species.

## 1 Introduction

Marine fish habitats are typically large, continuous, and lack definitive boundaries. Fishes that inhabit the epipelagic zone are generally less taxonomically diverse than species found in other habitats (benthic, coastal, reef‐associated, estuarine), possibly because the overall homogeneity of epipelagic habitats may reduce rates of speciation (Hamner, 1995). Nevertheless, some widespread and diverse fish families such as scombrids, belonids, hemiramphids, and exocoetids have circumtropical distributions that include a diversity of habitats (Gaither et al., 2015). The underlying mechanisms responsible for diversification in these fishes remain unclear, at least in part because their phylogenetic relationships are poorly resolved and life history characteristics little known. Phylogenetic characterizations are necessary to understand speciation because they define the sequence of lineage and species diversification. Also, phylogenies can clarify species identity when taxa are morphologically very similar (cryptic species), thereby improving understanding of species geographic distributions (Bass et al., 2005; Colborn et al., 2001; Quattro et al., 2005).

**Figure 1:**
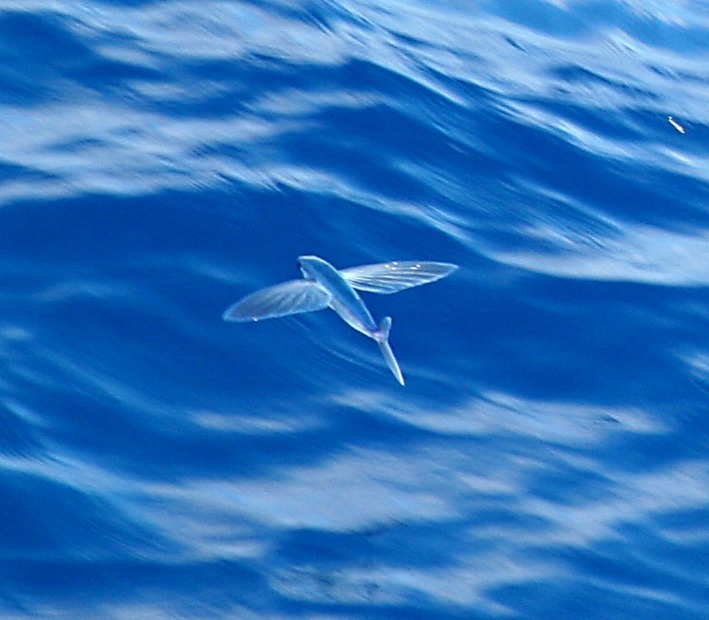
An *Exocoetus* fish gliding along the surface of epipelagic water in the eastern tropical Pacific. Photo credit: EAL (first author)

**Figure 2:**
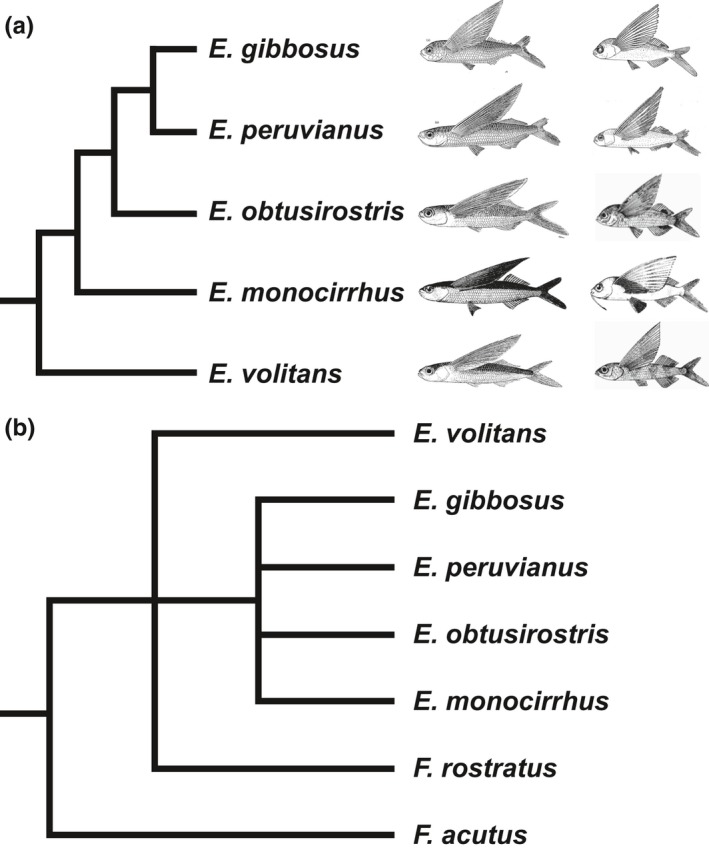
Morphology‐based phylogenetic hypotheses for *Exocoetus*. (a) Phylogenetic hypothesis presented by Parin and Shakhovskoy (2000). Illustrations of adults and juveniles were compiled from the following publications: *Exocoetus volitans* (Parin, 2002), *Exocoetus obtusirostris* (Parin, 2002), *Exocoetus monocirrhus* adult (Parin, 1984); *Exocoetus monocirrhus* juvenile (Heemstra & Parin, 1986), *Exocoetus peruvianus* (Parin & Shakhovskoy, 2000), *Exocoetus gibbosus* (Parin & Shakhovskoy, 2000). (b) Phylogenetic hypothesis using the same 11 morphological characters as Parin and Shakhovskoy (2000), with added morphological data from outgroups *Fodiator acutus* and *Fodiator rostratus*; strict consensus of 4 equally parsimonious trees of 12 steps each

Comprehensive species phylogenies can provide key insights regarding speciation in marine lineages with high dispersal potential, wide ranges, and overlapping distributions. *Exocoetus* (two‐wing flyingfish) is a monophyletic genus of five species found in the epipelagic waters of tropical and subtropical oceans worldwide (Lewallen et al., 2011; Parin & Shakhovskoy, 2000). Gliding on elongated pectoral fins (Figure 1) separates *Exocoetus* from most other members of the family Exocoetidae that can use elongated pectoral, pelvic, and sometimes dorsal fins to achieve prolonged aerial glides. As with many widely distributed fishes, *Exocoetus* has buoyant, pelagic eggs, and larvae that persist in the epipelagic zone during maturation, which occurs at lengths of 130–155 mm (SL) (Grudtsev et al., 1987). *Exocoetus* individuals live for approximately 1 year, are small [max SL ≤ 207 mm (Grudtsev et al., 1987)], slow swimming, and incapable of long‐distance migrations (Parin, 1968). Curiously, the distribution of each *Exocoetus* species overlaps with at least one other species, suggesting they may have evolved in parapatry or sympatry. Species ranges vary from circumtropical (e.g., *E. volitans*) to single oceanographic regions (e.g., *E. peruvianus*), indicating differences in habitat specialization. Although three species of *Exocoetus* were traditionally recognized [*E. volitans*,* E. monocirrhus*,* E. obtusirostris* (Kovalevskaya, 1982; Parin, 1961)], two cryptic species previously grouped within *E. obtusirostris* have been described more recently (*E. peruvianus* and *E. gibbosus*) (Lewallen et al., 2011; Parin & Shakhovskoy, 2000). A phylogenetic hypothesis for *Exocoetus* (Parin & Shakhovskoy, 2000) that was previously proposed based on 11 morphological characters (Figure 2a) has not been tested using strict inference methods or molecular data.

A thorough examination of genetic diversity in *Exocoetus* is greatly needed, considering the potential for uncovering cryptic species (especially within the globally distributed *E. volitans*). Here, through extensive sampling and phylogenetic analysis, we improve the resolution of evolutionary lineages within *Exocoetus*, thereby providing new data on how speciation occurs in the epipelagic zone. We specifically focused on the following questions: (1) What are the phylogenetic relationships within *Exocoetus* based on molecular data, and how do they compare to the most recent morphological hypothesis? (2) Do the currently recognized *Exocoetus* species represent distinct monophyletic lineages, and are there cryptic species? (3) What biogeographic patterns of speciation are revealed by phylogenetic arrangements within this genus?

## 2 Materials and Methods

### 2.1 Taxon sampling

A total of 429 flyingfish specimens (422 *Exocoetus* and seven outgroup specimens) were collected at night using long‐handled dipnets and/or donated by collaborators (Appendix S1). Animals were euthanized in an ice‐water bath. Post‐mortem handling included shipboard freezing in seawater, removal of lateral muscle tissue for DNA analysis (95% ethanol), whole‐specimen fixation (10% formalin), and long‐term museum archiving (70% ethanol). Each specimen was identified using key diagnostic characters (e.g., gill raker counts and body depth measurements) as presented in (Parin & Shakhovskoy, 2000). All voucher specimens are archived with catalogue numbers at the Royal Ontario Museum or Scripps Institution of Oceanography (Appendix S1). We note that 15 specimens used in the current study were included in a previous study (Lewallen et al., 2011). Also, 266 *E. volitans* specimens were sequenced (Cytb) for a previous population genetic analysis (Lewallen et al., 2016). Details regarding which specimens are common among studies are provided in Appendix S1.

### 2.2 Morphological data

Parin and Shakhovskoy (2000) presented a series of morphological characters for *Exocoetus*, and a phylogenetic hypothesis for the genus (Figure 2a). However, their study used dichotomous morphological character analyses to discern species rather than explicit phylogenetic analyses. Importantly, only 11 characters in Parin & Shakhovskoy's study were informative for distinguishing species and could be clearly coded for phylogenetic analysis. To test the morphology‐based phylogeny for this genus, we tabulated the characters presented in Parin and Shakhovskoy (2000) into a data matrix (Table 1). Data for the 11 characters for two outgroup taxa (*Fodiator acutus* and *F. rostratus*) were obtained from the literature (Parin & Belyanina, 2002; Parin & Shakhovskoy, 2000; Table 1).

**Table 1:** Morphological character matrix for the 11 characters described by Parin and Shakhovskoy (2000). Characters were coded as binary (1 or 0). Outgroup taxa (*Fodiator acutus* and *F. rostratus*) were added to this matrix using morphological data presented by Parin and Belyanina (2002) and Parin and Shakhovskoy (2000)

	Morphological characters										
	1	2	3	4	5	6	7	8	9	10	11
*E. volitans*	0	0	0	0	0	1	1	1	0	0	0
*E. monocirrhus*	1	1	1	1	1	0	0	0	1	0	0
*E. obtusirostris*	1	1	1	1	1	0	1	1	0	0	1
*E. peruvianus*	1	1	1	1	1	0	1	1	0	1	0
*E. gibbosus*	1	1	1	1	1	0	1	1	1	1	0
*F. acutus*	0	0	0	0	0	0	1	1	0	0	0
*F. rostratus*	0	0	0	0	0	0	1	1	0	0	0

*Character Coding*: 1, Supraoccipital with one posterior process; 2, Presence of posteromedian process on cleithrum; 3, Humpbacked juveniles; 4, Ventral fins are anteriorly shifted; 5, Increased number of scales in transverse row; 6, Shortened ventral fins; 7, Jaw teeth much reduced; 8, Absence of barbel in juveniles; 9, High body depth in juveniles; 10, Maximum development of posterolateral process on cleithrum; 11, High number of rays in pectoral fin.

### 2.3 Molecular data

For phylogenetic analysis of *Exocoetus*, mitochondrial encoded cytochrome b gene (Cytb; 1,082 bps) and nuclear recombination activating gene 2 (Rag2; 882 bps) sequence data were obtained for 14 individuals including 2 representatives of each *Exocoetus* species and 4 outgroup specimens (*Cheilopogon xenopterus*,* F. rostratus*,* Hirundichthys marginatus*,* Parexocoetus brachypterus*; Table 2). To test for cryptic speciation, we generated an expanded molecular dataset by collecting mitochondrial sequence data (Cytb) for 422 *Exocoetus* specimens (266 *E. volitans*, 9 *E. peruvianus*, 2 *E. gibbosus*, 9 *E. obtusirostris*, and 136 *E. monocirrhus*), and 4 outgroup specimens (2 *P. hillianus* and 2 *P. brachypteru*s). The globally distributed *E. volitans* was collected from the Atlantic (*n* = 150), Pacific (*n* = 111), and Indian (*n* = 5) Oceans. For *E. monocirrhus*, we collected individuals from the eastern and central Pacific (*n* = 131), as well as Indian (*n* = 5) Ocean. *E. obtusirostris* specimens (*n* = 9) were obtained from the Atlantic Ocean and Gulf of Mexico. Because they have restricted distributions, only small numbers of *E. peruvianus* (*n* = 9) and *E. gibbosus* (*n* = 2) were obtained from waters of the Peruvian Upwelling Current and South Pacific Subtropical Gyre, respectively.

**Table 2:** *Exocoetus* specimens with cytochrome b (Cytb) and recombination activating gene 2 (Rag2) sequence data. Ingroup and outgroup taxa are specified and voucher catalogue numbers, collection localities, Genbank accession numbers, and citations are listed

Specimen data					Genbank accession number		
Genus	Species	Specimen No.	Voucher No.	Locality	Cytb	Rag2	Citation
*Exocoetus*	*gibbosus*	3717	ROM‐79289	ETP	KY382508	KY385897	This study
*Exocoetus*	*gibbosus*	4568	ROM‐92584	ETP	KY382509	KY385898	This study
*Exocoetus*	*monocirrhus*	1572	SIO‐07‐129	ETP	HQ325628	HQ325695	Lewallen et al. (2011)
*Exocoetus*	*monocirrhus*	5801	ROM‐79270	ETP	HQ325629	HQ325696	Lewallen et al. (2011)
*Exocoetus*	*obtusirostris*	1851	NMNH380590	Atlantic	HQ325630	HQ325697	Lewallen et al. (2011)
*Exocoetus*	*obtusirostris*	1854	NMNH380574	Atlantic	HQ325631	HQ325698	Lewallen et al. (2011)
*Exocoetus*	*peruvianus*	1611	SIO‐07‐125	ETP	HQ325632	HQ325699	Lewallen et al. (2011)
*Exocoetus*	*peruvianus*	1612	SIO‐07‐125	ETP	HQ323633	HQ325700	Lewallen et al. (2011)
*Exocoetus*	*volitans*	1585	SIO‐07‐132	ETP	HQ325634	HQ325701	Lewallen et al. (2011)
*Exocoetus*	*volitans*	1586	SIO‐07‐132	ETP	HQ325635	HQ325702	Lewallen et al. (2011)
*Cheilopogon*	*xenopterus*	3785	ROM‐79248	ETP	HQ325621	HQ325688	Lewallen et al. (2011)
*Fodiator*	*rostratus*	1570	SIO‐07‐128	ETP	HQ325638	HQ325705	Lewallen et al. (2011)
*Hirundichthys*	*marginatus*	3181	ROM‐79330	ETP	HQ325644	HQ325711	Lewallen et al. (2011)
*Parexocoetus*	*brachypterus*	4148	ROM‐79331	ETP	HQ325656	HQ325723	Lewallen et al. (2011)

ETP, Eastern Tropical Pacific.

Genomic DNA was extracted using DNeasy kits (Qiagen, Valencia, CA, USA). A portion of both the Cytb and Rag2 genes were amplified using previously published primers ExoCBFwd, ExoCBRev, and Ffly‐Ch, Rfly‐Ch, respectively (Lewallen et al., 2011). One advantage of using Rag2 over some other nuclear genes is that it does not contain introns in the coding region (Peixoto, Mikawa, & Brenner, 2000). PCR conditions, internal sequencing primers (ExoFwd1 and ExoRev1 for Cytb; F16‐Ch and R17‐Ch for Rag2), and sequence alignment methods followed Lewallen et al. (2011).

### 2.4 Maximum Parsimony

Maximum parsimony (MP) analyses were conducted on the following five datasets using PAUP* 4.10b (Swofford, 2000). Morphological and genetic data were concatenated using MacClade 4.07 (Maddison & Maddison, 2005): *Set 1*: morphological data, 7 taxa, 11 characters (Parin & Belyanina, 2002; Parin & Shakhovskoy, 2000; Table 1); *Set 2*: Cytb data, 14 specimens, 1,137 bps each (Table 2); *Set 3*: Rag2 data, 14 specimens, 882 bps each (Table 2); *Set 4*: All data combined, 14 specimens, morphology, Cytb, Rag2 (Tables 1 and 2); *Set 5*: An expanded Cytb dataset, 427 specimens, 1,082 bps each (Appendix S1). Morphological data (Set 1) were analyzed using the exhaustive search algorithm (MP) within PAUP* 4.10b (Swofford, 2000). *Fodiator acutus* and *Fodiator rostratus* were defined as outgroup taxa and a strict consensus of the four most parsimonious trees was generated.

For MP analysis of Cytb (Set 2) and Rag2 (Set 3) data, we used heuristic searches (10,000 random addition sequence replicates and TBR branch swapping). *Cheilopogon xenopterus*,* Hirundichthys marginatus*,* Parexocoetus brachypterus*, and *Fodiator rostratus* were defined as outgroups, and strict consensus trees were calculated. Support for nodes was measured by performing 100 bootstrap replicates (BS), with 10,000 random addition sequence replicates per bootstrap iteration. For the combined analysis (Set 4), *C. xenopterus*,* H. marginatus*,* P. brachypterus*, and *F. rostratus* were defined as outgroup taxa. A heuristic search using 10,000 random addition sequence replicates and TBR branch swapping was performed. Bootstrap support was calculated with 100 bootstrap replicates and 10,000 random addition sequence replicates per bootstrap iteration. The expanded Cytb dataset (Set 5) was analyzed using a heuristic search of 1,000 random addition sequence replicates, and TBR branch swapping. For this analysis, four non‐*Exocoetus* sequences were included (2 *P. hillianus* and 2 *P. brachypterus*) and designated as outgroups (Appendix S2).

### 2.5 Bayesian inference

We analyzed Sets 2 through 5 (see above) using Bayesian inference (BI) implemented by BEAST 1.6.1 (Drummond & Rambaut, 2007). Outgroup taxa for each dataset were the same as in MP analyses above. As in previous phylogenetic analyses of these taxa and molecular markers (Lewallen et al., 2011), a general time reversible model with invariant sites and gamma distribution (GTR + I + Γ) was determined as the best model of evolution, and was used for this study. Using a random starting tree, 10 million MCMC generations were run, saving one of every 1,000 trees, and the first 10% of saved trees were discarded as burn‐in. TRACER 1.4 (Rambaut & Drummond, 2007a) was used to view the posterior distribution of sampled trees and assess convergence, and TreeAnnotator 1.4 (Rambaut & Drummond, 2007b) was used to calculate a maximum clade credibility tree. Phylograms were generated using TreeView (Page, 1996), with branch lengths corresponding to substitutions per site and Bayesian posterior probabilities (BPP) presented at each node.

### 2.6 Genetic distance

To estimate genetic distances among sampled individuals, mean Kimura two‐parameter (K2P) values (Kimura, 1980) were calculated using MEGA 5 (Tamura et al., 2011). All possible pairwise comparisons were calculated among individuals within each species, and also between each species. Between‐species genetic distance estimates were then used to obtain an overall mean for the genus.

## 3 Results

### 3.1 Maximum parsimony

**Figure 3:**
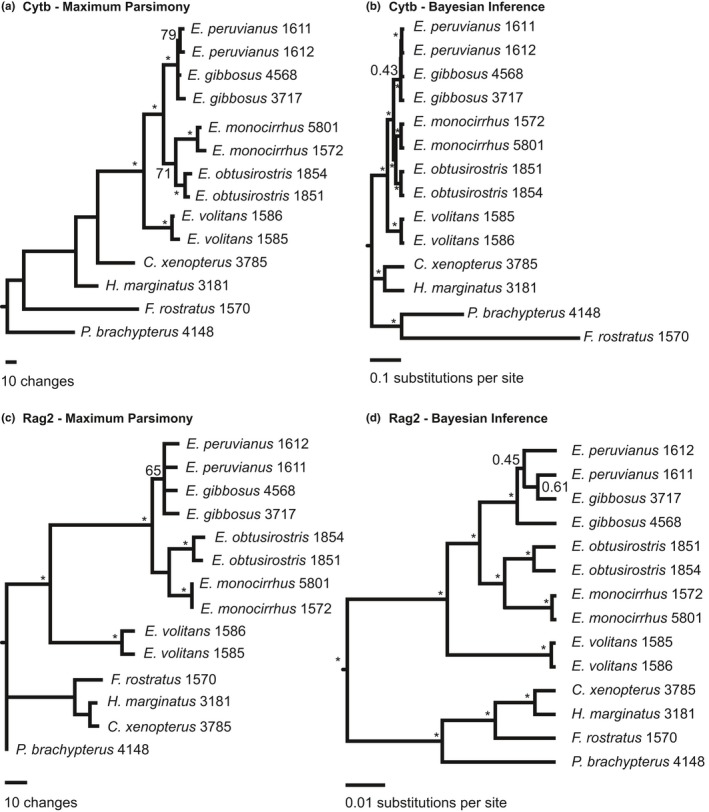
Phylogenetic analyses of *Exocoetus* specimens using each gene (Cytb = cytochrome b, Rag2 = recombination activating gene 2) and inference method (MP = maximum parsimony using PAUP* 4.10b (Swofford, 2000), BI = Bayesian inference using BEAST 1.6.1 (Drummond & Rambaut, 2007)). Bootstrap proportions (BS) and Bayesian posterior probabilities (BPP) are listed above nodes. *Cheilopogon xenopterus*,* Hirundichthys marginatus*,* Parexocoetus brachypterus*, and *Fodiator rostratus* were used as outgroup taxa. (a) MP analysis of Cytb data, (b) BI analysis of Cytb data, (c) MP analysis of Rag2 data, (d) BI analysis of Rag2 data

**Figure 4:**
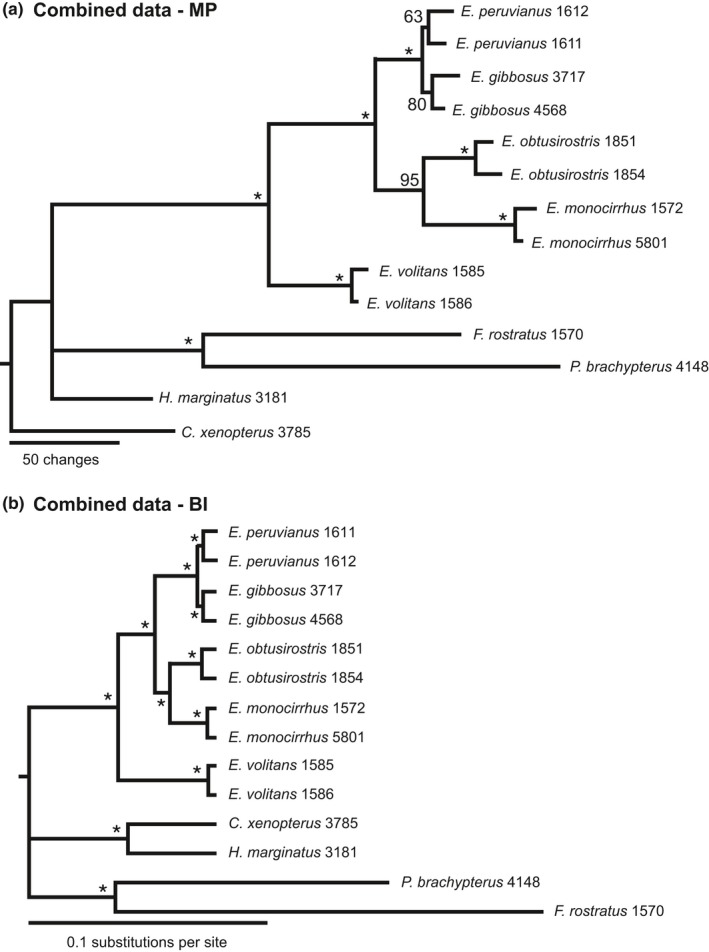
Combined evidence phylogenies analyzed using maximum parsimony (MP) and Bayesian inference (BI) methods and 2,030 total characters (Cytb = 1,137, Rag2 = 882, morphology = 11). *Cheilopogon xenopterus*,* Hirundichthys marginatus*,* Parexocoetus brachypterus* and *Fodiator rostratus* were used as outgroup taxa. (a) MP phylogeny with bootstrap proportions (BS) listed next to each node (*100). (b) BI phylogeny with branch lengths corresponding to the number of base pair differences between sequences. *indicates Bayesian posterior probabilities ≥0.96

Our MP analysis of the 11 morphological characters (Set 1) used by Parin and Shakhovskoy (Parin & Shakhovskoy, 2000) provided limited phylogenetic resolution (Figure 2b). The tree contains a polytomy of four *Exocoetus* species (*E. gibbosus*,* E. peruvianus*,* E. obtusirostris*, and *E. monocirrhus*), and this lineage is placed in an unresolved polytomy with *E. volitans* and *F. rostratus* (Figure 2b). Our MP analyses that included molecular data for 14 individuals (Sets 2–4) support the monophyly of *Exocoetus* (Figures 3a,c, 4a). These analyses supported *E. volitans* as the sister taxon to all other *Exocoetus* species with BS ≥ 98. The arrangement of *E. monocirrhus* as sister to an *E. obtusirostris* – *E. peruvianus* – *E. gibbosus* clade proposed by Parin and Shakhovskoy (Parin & Shakhovskoy, 2000) (Figure 2a) is not supported by any of the MP analyses conducted here, including analysis involving morphological characters (Figures 2b, 3a,c, 4a). Similarly, the grouping of *E. obtusirostris* as sister to *E. gibbosus* – *E. peruvianus* (Figure 2a) is also not supported (Figures 2b, 3a,c, 4a). Instead, our analyses show sister clades of *E. monocirrhus* – *E. obtusirostris* and *E*. *peruvianus* – *E. gibbosus* with high support (BS ≥ 98) in the combined analysis (Figure 4a), and independent Cytb (Figure 3a) and Rag2 (Figure 3c) phylogenies.

The monophyletic grouping of multiple representatives of each species was generally observed in the Cytb (Figure 3a, BS ≥ 79) and combined analyses (Figure 4a, BS ≥ 63). However, analysis of Rag2 data did not resolve *E. gibbosus* and *E. peruvianus* individuals as distinct species (Figure 3c), and *E. gibbosus* individuals were not monophyletic in the Cytb analysis (Figure 3a).

Maximum parsimony analysis of the expanded Cytb dataset (Set 5) including 426 individuals (1,082 total bps, 319 parsimony informative) grouped multiple individuals of *E. volitans*,* E. obtusirostris*, and *E. monocirrhus* into monophyletic clades corresponding to species, but did not clearly segregate *E. peruvianus* from *E. gibbosus* (Appendix S2). Rather, *E. gibbosus* individuals were nested within *E*. *peruvianus*. This topology shows *E. volitans* as sister to all other *Exocoetus* species, and *E. monocirrhus* and *E. obtusirostris* as sister taxa.

### 3.2 Bayesian Inference

Bayesian inference analyses of Sets 2, 3, and 4 produced topologies similar to MP analyses, and consistently supported *E. volitans* as sister to all other *Exocoetus* species (BPP ≥ 0.96, Figures 3b,d, 4b). As in MP results, *E. monocirrhus* and *E. obtusirostris* are sister taxa in every analysis, with strong support (BPP ≥ 0.96). The arrangement of *E. peruvianus* and *E. gibbosus* individuals in a monophyletic clade was well supported across analyses, but individuals from each of these species did not form monophyletic groups in the Rag2 analysis (Figure 3d). We did not find evidence for the phylogenetic arrangement of *E. monocirrhus* as sister to an *E. obtusirostris–E. peruvianus–E. gibbosus* clade in any of the BI analyses.

The BI analysis of the expanded Cytb dataset (Set 5) resulted in well‐supported clades (BPP ≥ 0.97) with *Exocoetus* monophyletic and *E. volitans* as the sister to all other species. A clade comprised of *E. peruvianus–E. gibbosus*, was sister to a clade comprised of *E. obtusirostris–E. monocirrhus*. Multiple representatives of *E. volitans*,* E. monocirrhus*, and *E. obtusirostris* grouped together in every analysis, whereas *E. gibbosus* and *E. peruvianus* were not clearly distinguished.

### 3.3 Genetic distance

**Figure 5:**
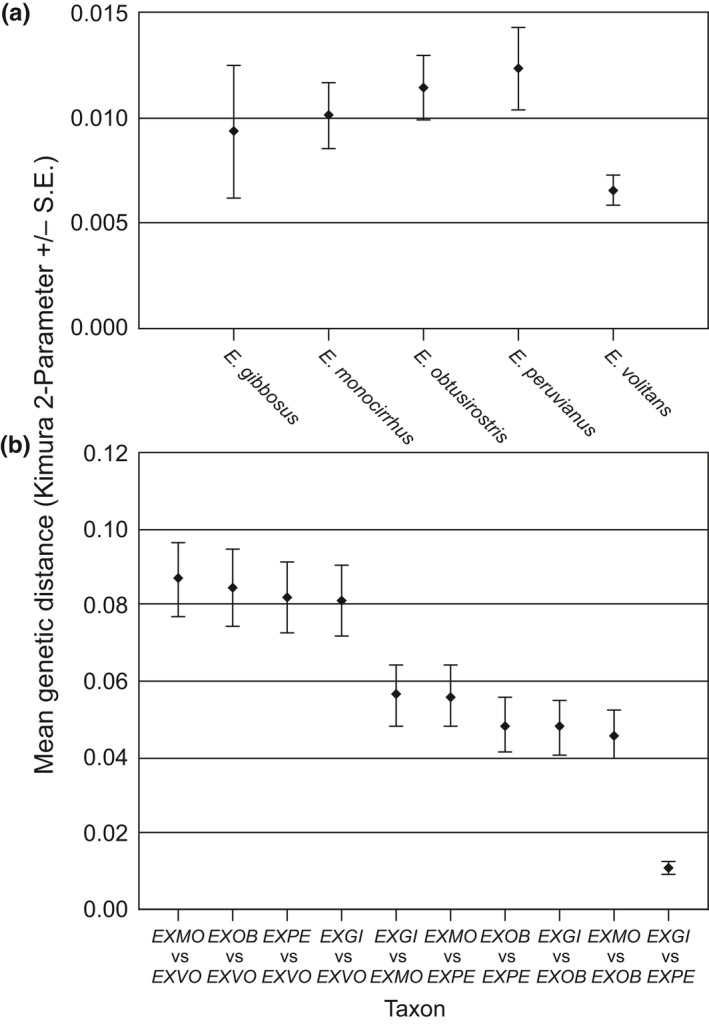
Summary of genetic distance estimates (Kimura two‐Parameter; K2P) within and between *Exocoetus* species based on the expanded Cytb dataset (*n* = 1,082 bps). (a) Mean Cytb genetic distances (K2P ± standard error; S.E.) within each species of *Exocoetus*. (b) Mean Cytb genetic distance estimates (K2P ± standard error; S.E.) between *Exocoetus* species. Species are abbreviated using the following labels: EXVO = *E. volitans*, EXMO = *E. monocirrhus*, EXOB = *E. obtusirostris*, EXPE = *E. peruvianus*, EXGI = *E. gibbosus*

Pairwise estimates of K2P genetic distances for Cytb between individuals within each *Exocoetus* species were 0.012 (±0.002) for *E. peruvianus*, 0.011 (±0.002) for *E. obtusirostris*, 0.010 (±0.002) for *E. monocirrhus*, and 0.007 (±0.001) for *E. volitans*. For *E. gibbosus*, the K2P genetic distance between the two individuals was 0.009. Genetic distance comparisons between species ranged from 0.011 (*E. gibbosus* vs. *E. peruvianus*) to 0.087 (*E. monocirrhus* vs. *E. volitans*), with an overall mean K2P value of 0.060 (Figure 5).

## 4 Discussion

### 4.1 Phylogeny of exocoetus

Phylogenetic analyses of *Exocoetus* species yielded consistent, well‐supported evidence for the arrangement of four monophyletic groups irrespective of the method used. First, *Exocoetus* is monophyletic, which corroborates the findings of other authors (Collette et al., 1984; Lewallen et al., 2011; Parin, 1961; Parin & Shakhovskoy, 2000). Second, *E. volitans* is sister to all other *Exocoetus* species, with *E. monocirrhus* + *E. obtusirostris* + *E. peruvianus* + *E. gibbosus* forming a monophyletic clade. Third, *E. monocirrhus* is sister to *E. obtusirostris*. Fourth, a monophyletic group containing *E. peruvianus* and *E. gibbosus* is well supported. Our results agree with the hypothesis of Parin and Shakhovskoy (2000), with the exception of several key relationships. We did not find evidence for an *E. obtusirostris* + *E. gibbosus* + *E. peruvianus* clade sister to *E. monocirrhus*. Additionally, none of our analyses yielded support for the arrangement of *E. obtusirostris* as sister to an *E. gibbosus–E. peruvianus* clade. Furthermore, in all analyses (except using morphological data only), we found support for a sister species relationship between *E. monocirrhus* and *E. obtusirostris*, which was not included in the Parin and Shakhovskoy (2000) hypothesis. Although there is support for a clade containing *E. peruvianus* and *E. gibbosus*, sequences from these species were not always reciprocally monophyletic, suggesting that they are not genetically distinct species, or that speciation has been rapid, recent and/or ongoing (see below).

### 4.2 Species distinctions

To assess the genetic distinctiveness of *Exocoetus* species, we performed a genetic survey of specimens collected worldwide and found that *E. volitans*,* E. monocirrhus*, and *E. obtusirostris* are distinct species that clearly form monophyletic groups. These three species can easily be distinguished using morphological or molecular characters. *E. gibbosus* and *E. peruvianus* comprise a well‐supported monophyletic group, yet the evolutionary separation of these species is less clear. These species are distributed allopatrically, with *E. peruvianus* found offshore of Peru, and *E. gibbosus* found in the South Pacific. The two species are therefore separated by the Eastern Pacific Barrier, a 4,000–7,000 km wide stretch of deep ocean without islands. However, despite this apparent allopatric distribution, phylogenetic and genetic distinctiveness of these taxa is lacking. Individuals were not reciprocally monophyletic by species, and genetic divergence was low (K2P = 1.1%). Morphological characters distinguishing *E. peruvianus* from *E. gibbosus* are subtle, involving proportional body form measurements. For example, in adults (>150 mm SL), the body depth at the pectoral fin base is 19–22% SL in *E. gibbosus* and 15–18.5% SL in *E. peruvianus*. Additionally, in juveniles (<80 mm SL), the head depth is 22.5–28.5% SL in *E. gibbosus*, and 19.5–23% in *E. peruvianus* (Parin & Shakhovskoy, 2000).

Two main situations could result in the lack of evidence for the taxonomic distinctiveness of *E. peruvianus* and *E. gibbosus*. One possibility is very recent speciation, accompanied by incomplete lineage sorting. Gene trees may not be congruent with a species tree when the rate of speciation exceeds the rate at which allelic polymorphisms achieve reciprocal monophyly in separated gene pools (Harrison, 1991). Although we have not calibrated a molecular clock for this study, the very low amounts of divergence between individuals of the two putative species are indicative of very recent divergence. A second possibility is that *E. peruvianus* and *E. gibbosus* represent a single species, with regular gene flow between two distant allopatric populations, sufficient to prevent them from becoming reproductively isolated. Observed morphological differences might be due to phenotypic plasticity associated with the occupation of slightly different habitats. If this is the case, these species would be better classified as regional morphotypes of the same species, a pattern that has been observed in other flyingfishes (Parin & Belyanina, 1998). Our results point to the need for additional sampling and genetic analyses to confirm whether *E. peruvianus* and *E. gibbosus* represent distinct species. Increasing the number of sampled individuals, or using higher‐resolution genetic markers would likely improve our ability to differentiate between the two scenarios described above. We also note that adding samples for lineages with lower numbers of individuals sequenced would reduce any possible biases caused by differences in sample number across species analyzed. For example, we are likely to have incompletely sampled the total Cytb variation of *E. peruvianus* and *E. gibbosus,* and further sequencing may provide clearer indication of whether these putative species are genetically isolated.

### 4.3 Cryptic species

Cryptic species (Bickford et al., 2007) have long posed taxonomic challenges and may be identified using anatomical, ecological, behavioral, biogeographic, and/or molecular characteristics. DNA comparisons can provide particularly useful information about species distinctiveness. Hebert et al. (2003) suggested that genetic distance estimates above 3% for the DNA “barcode” gene cytochrome oxidase I should be used as a threshold for defining species, and genetic distance estimates above 2% have been proposed for distinguishing vertebrate species using Cytb data (Avise & Walker, 1999). However, other studies have shown that model selection for genetic distance calculations can also affect species delimitation (Barley & Thomson, 2016). In our study, very low mean Cytb genetic distance estimates (K2P = 0.7–1.2%) within each species suggests an absence of cryptic species. In addition, in the case of *E. volitans*, an analysis across the range of the species found minimal population genetic structure at a global scale (Lewallen et al., 2016).

Single widely distributed marine taxa are sometimes found to consist of morphologically cryptic, but genetically distinct, independent evolutionary lineages (species) segregated by ocean basin (Briggs, 1960), or oceanographic factors (Gaither et al., 2015). Examples include bristlemouths (Miya & Nishida, 1997), goliath groupers (Craig et al., 2009), bonefish (Bowen, Karl, & Pfeiler, 2007), ocean sunfish (Bass et al., 2005), and hammerhead sharks (Quattro et al., 2005). In contrast to these documented cases of cryptic species, we find no evidence of this phenomenon in *Exocoetus,* despite the multi‐ocean distributions of *E. volitans* and *E. monocirrhus*. At least some globally connected species maintain global population connectivity by dispersal (e.g., pelagic wahoo; Theisen et al., 2008). For *Exocoetus*, buoyant pelagic eggs likely provide an adequate mechanism for dispersal across large distances. The exact pelagic larval duration of *Exocoetus* is not known, but Mora et al. (2012) recently demonstrated that the larvae of many tropical reef fishes persist in the water column long enough for regular breaching of the Eastern Pacific Barrier. Thus, long‐distance dispersal of pelagic eggs could explain the lack of genetic differentiation of *Exocoetus* populations from different Oceans.

### 4.4 Exocoetus biogeography

**Figure 6:**
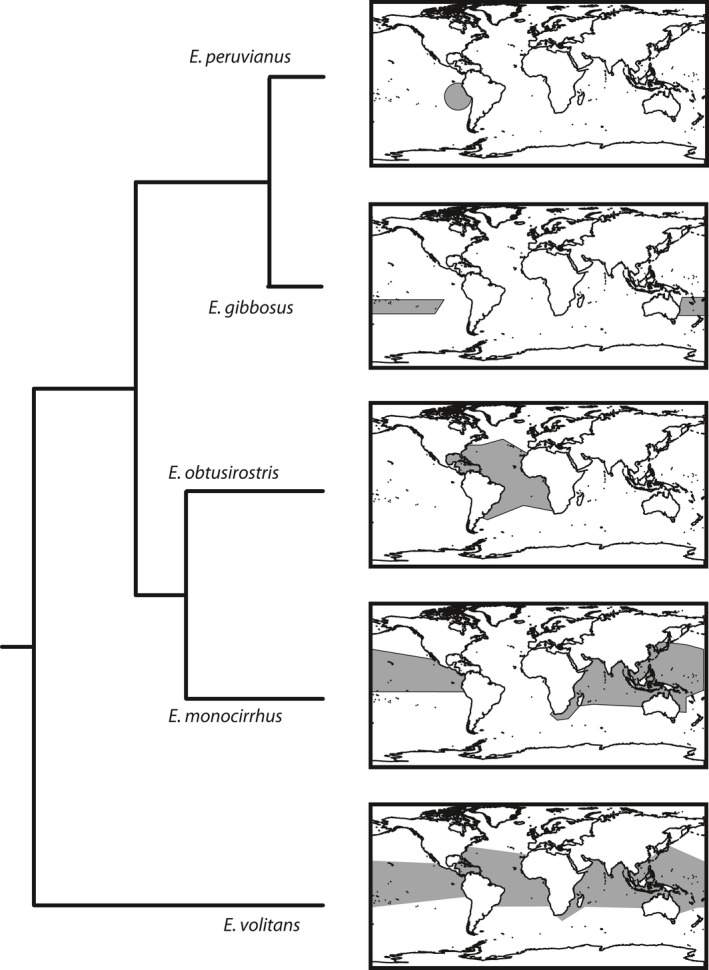
Phylogeny of *Exocoetus* with distribution maps derived from collection localities presented in Parin & Shakhovskoy (Parin & Shakhovskoy, 2000). Polygons were produced using ArcMap 9.3.1 (ESRI, Redlands, CA, USA)

At the base of the *Exocoetus* tree, *E. volitans* is distributed throughout all tropical oceans and sympatric with every other species in the genus (Figure 6) although granular patterns of habitat preference might preclude contact among individuals from different species (e.g., seasonally sympatric/parapatric). As sister to all other species in this genus, we conclude that the ancestor of *Exocoetus* fishes may have been similar to *E. volitans*, both in terms of morphology and distribution. The distribution of the sister lineage to *E. volitans* may also have had an expansive distribution, so inferring the geographic context of divergence within *Exocoetus* is difficult. Sympatric diversification between two globally distributed lineages is a possibility, but equally realistic is allopatric diversification followed by significant dispersal and range expansion.

Because *E. volitans* individuals from the Atlantic and Indo‐Pacific are not genetically diverged (Lewallen et al., 2016), we suggest that the species either dispersed between these regions very recently, or that there is regular gene flow between Oceans, presumably across the Benguela Barrier. The Benguela Barrier results from the upwelling of cold waters near the tip of South Africa and can prevent dispersal between the tropical Atlantic and tropical Indian Oceans for some marine fishes (Briggs, 1995; Rocha, Craig, & Bowen, 2007). However, Rocha et al. (2005) provided compelling evidence to suggest that at least some tropical marine fishes (*Gnatholepis* gobies) have breached the Benguela Barrier to invade the Atlantic Ocean from the Indian Ocean. Additionally, Craig, Hastings and Pondella (2004) showed support for a sister species relationship between Caribbean and Western Indian Ocean species of the grouper genus *Dermatolepis*, demonstrating trans‐Atlantic dispersal and crossing of the Benguela Barrier by reef fishes (Craig et al., 2004). The distribution of *E*. *volitans*, and the minimal genetic divergence between individuals from the Atlantic and Indian Oceans suggest that *Exocoetus* may be capable of similar dispersals.

The two most distal nodes of the *Exocoetus* tree (*E. obtusirostris* + *E. monocirrhus* and *E. peruvianus* + *E. gibbosus*) provide better opportunities for determining the biogeographic context of diversification (Figure 6). In particular, previously identified marine biogeographic barriers (Rocha et al., 2007) are relevant to the phylogenetic and geographic patterns we observe in *Exocoetus*. In the case of the sister relationship between *E. monocirrhus* and *E. obtusirostris*, their respective distributions in the Indian and Pacific Oceans versus the Atlantic Ocean suggest that the Isthmus of Panama and Benguela Barriers may have provided effective boundaries to limit gene flow, resulting in speciation. On the west side of the Atlantic Ocean, the Isthmus of Panama is a well‐known land barrier that has resulted in the speciation of many Atlantic and Pacific sister lineages of marine fishes (Banford, Bermingham, & Collette, 2004). According to Banford et al. (2004), at least four periods in the last 10 million years provided marine fishes with opportunities for allopatric speciation on opposite sides of the Isthmus of Panama as it gradually formed. Perhaps a result of species‐specific thermal tolerances, the cold waters of South Africa (Benguela Barrier) seem to effectively confine *E. obtusirostris* and *E. monocirrhus* to their respective tropical Atlantic and tropical Indo‐Pacific distributions.

The sister relationship between *E. peruvianus* and *E. gibbosus*, in combination with their respective distributions in the Peruvian Upwelling Current and South Pacific Subtropical Gyre (Parin & Shakhovskoy, 2000), suggests that the Eastern Pacific Barrier (Lessios & Robertson, 2006) may segregate these putative species. The Eastern Pacific Barrier is an expanse of deep water (4,000–7,000 km wide) that separates coastally distributed fishes, although Lessios and Robertson (2006) showed examples of species that can cross this barrier. The lack of islands in the eastern Pacific makes it a particularly effective barrier for some reef‐inhabiting organisms. However, this barrier should, in principle, only influence coastal or neritic flyingfish species (e.g., *Fodiator*,* Parexocoetus*), or species that have island‐associated life stages (e.g., *Cheilopogon atrisignis*,* Cypselurus angusticeps*), and would be expected irrelevant to holoepipelagic species, such as *Exocoetus*. Thus, *E. peruvianus* and *E. gibbosus* may be separated by some other oceanographic barrier. Additional samples are required to first determine whether these taxa are distinct, and then reveal if and how gene flow occurs across this putative barrier. Given the low amount of sequence divergence between *E. peruvianus* and *E. gibbosus*, we favor the hypothesis that this species pair is the result of recent divergence and provides a rare example of incipient speciation in an epipelagic fish lineage. As such, these taxa are good candidates for addressing the mechanisms by which speciation occurs in the epipelagic zone.

## Conflict of Interest

None declared.
