# Supplementary material for: Phylogenetics and biogeography of the two‐wing flyingfish (Exocoetidae: Exocoetus)
Source: Ecol Evol. 2017 Feb 12;7(6):1751–61. doi: 10.1002/ece3.2786 (PMC5355192; doi:10.1002/ece3.2786)
Supplement: Supplementary file 2 [file ECE3-7-1751-s002.pdf]

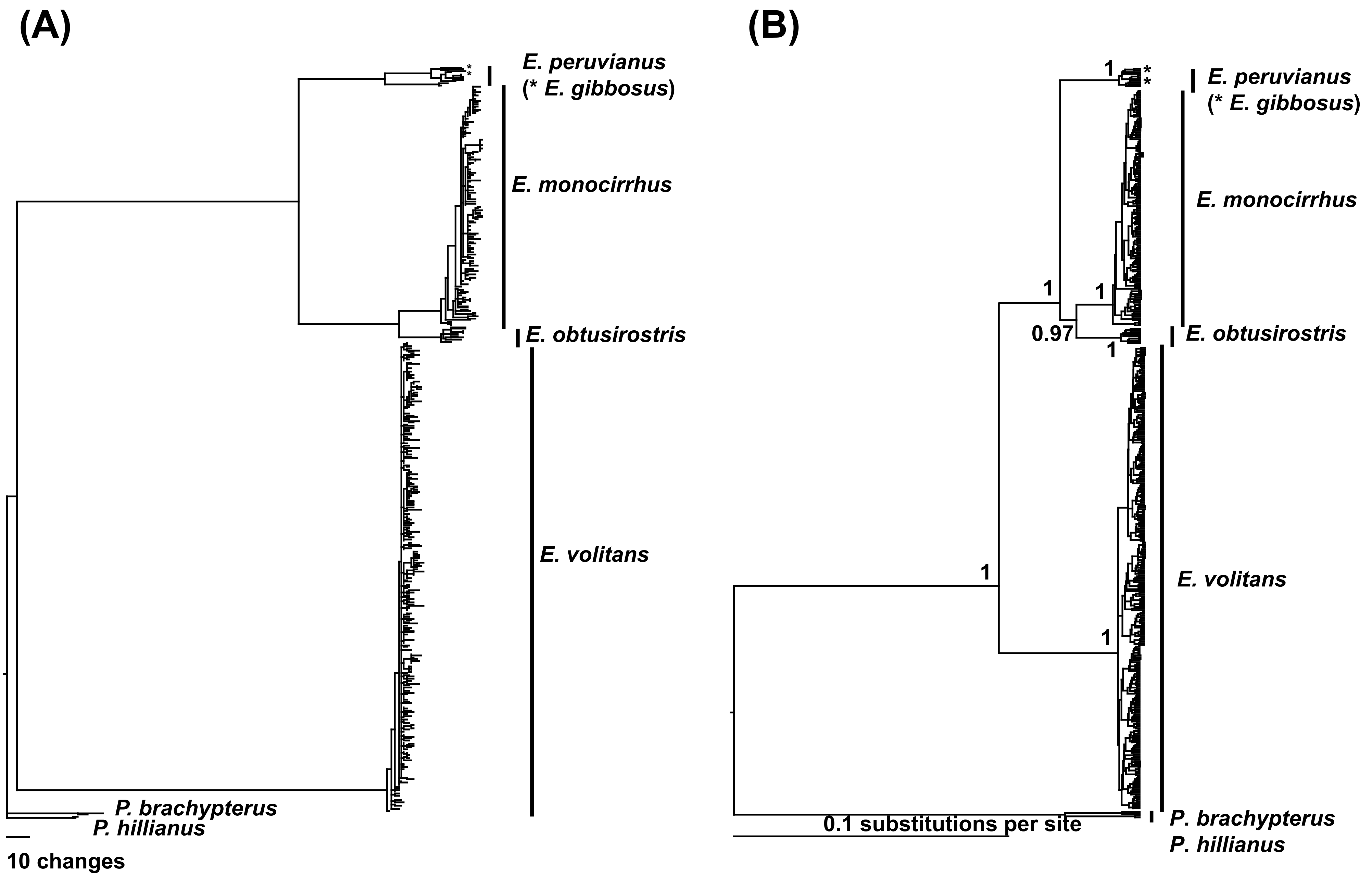
(A)
E. peruvianus
(* E. gibbosus)
E. monocirrhus
E. obtusirostris
E. volitans
P. brachypterus
P. hillianus
10 changes
(B)
E. peruvianus
(* E. gibbosus)
E. monocirrhus
E. obtusirostris
E. volitans
P. brachypterus
P. hillianus
0.1 substitutions per site
1
1
1
0.97
1
1
1
